# Harmony Search Optimisation of Dispersed Laminated Composite Plates

**DOI:** 10.3390/ma13122862

**Published:** 2020-06-26

**Authors:** Celal Cakiroglu, Gebrail Bekdaş, Zong Woo Geem

**Affiliations:** 1 Department of Civil Engineering, Turkish-German University, Sahinkaya Cad 86, Istanbul 34820, Turkey; 2 Department of Civil Engineering, Istanbul University, Cerrahpaşa, Istanbul 34310, Turkey; bekdas@istanbul.edu.tr; 3 College of IT Convergence, Gachon University, Seongnam 13120, Korea

**Keywords:** meta-heuristic optimisation, harmony search, laminated composite plates, buckling

## Abstract

One of the major goals in the process of designing structural components is to achieve the highest possible buckling load of the structural component while keeping the cost and weight at a minimum. This paper illustrates the application of the harmony search algorithm to the buckling load maximisation of dispersed laminated composite plates with rectangular geometry. The ply thicknesses and fiber orientation angles of the plies were chosen as the design variables. Besides the commonly used carbon fiber reinforced composites, boron/epoxy and glass/epoxy composite plates were also optimised using the harmony search algorithm. Furthermore, the optimisation algorithm was applied to plates with three different aspect ratios (ratio of the longer side length to the shorter side length of the plate). The buckling loads of the plates with optimised dispersed stacking sequences were compared to the buckling loads of plates with the commonly applied 0°, ±45°, and 90° fiber angle sequence and identical ply thicknesses. For all three aspect ratios and materials in this study, the dispersed stacking sequences performed better than the plates with regular stacking sequences.

## 1. Introduction

Fiber reinforced composite materials are increasingly being used in structural engineering because of their superior strength and stiffness properties compared with more conventional structural materials. Laminated composite plates are a type of structural member that are made of a number of layers with different fiber orientations. The sequence of fiber angles and ply thicknesses in laminated composite plates largely determines the performance of these structural members [1,2,3,4]. There has been extensive research on the optimisation of laminated composite plates to obtain a maximum performance from these structural members, while reducing their weight as much as possible. One of the major objectives in the optimisation of the structural performance is to maximise the buckling load of the plate, which we also deal with in this article.

Barakat et al. [5] investigated the optimum laminate configuration for boron/epoxy and carbon/epoxy laminated composite plates to achieve a maximum buckling load. They optimised the plate thickness by changing the layer thicknesses and orientations. For the optimisation, a sequential linear programming method was used. In the earlier literature in this field, mainly gradient-based methods and nonlinear programming (NLP) techniques were used in the optimisation process. Furthermore, the ply orientations were mostly fixed at 0°, ±45°, and 90° [6]. Conceicao et al. [7] aimed at minimising the weight of a laminated plate using a combination of sensitivity analysis and mathematical programming. In the literature of more recent years, genetic and metaheuristic algorithms are increasingly being used instead of linear programming techniques. A comprehensive study of optimisation methods used in the stacking sequence optimisation of laminated composite plates can be found in Ghiasi et al. [8]. Almeida [9,10] applied the harmony search algorithm and genetic algorithms in the design optimisation of laminated composites. As the objective, the buckling load of a symmetric laminated plate was maximized [9], while the weight and deflection of the plate under transverse distributed loading were minimised. In the literature [10], multi-objective minimisation for weight and deflection under transverse loading was carried out using genetic algorithms. In one of the earliest works using genetic algorithms in the optimisation of laminated plates, Potgieter and Stander [11] minimized the bending strain energy of a laminated composite plate under central point load and uniformly distributed loading. More recent articles using the genetic algorithm for the optimisation of laminated composites [12,13,14] can be counted. Another set of metaheuristic algorithms used in the optimisation of laminated composite plates is the ant colony algorithm and its variations. Abachizadeh et al. [15,16] used the continuous ant colony algorithm for the multi-objective optimisation of symmetric hybrid laminates comprising high stiffness graphite/epoxy and low stiffness glass/epoxy. The composite’s fundamental frequency was maximised, and its cost was minimised. Wang et al. [17] used a modified ant colony algorithm in order to maximize the buckling load of a laminated rectangular plate using a modified ant colony algorithm. Studies aiming to maximise the buckling load and stiffness of a laminated composite using the ant colony algorithm were published by Aymerich and Serra [18,19]. In these studies, the ply thicknesses were assumed to be constant and the fiber orientations were limited to angles of 0°, ±45°, and 90°. Sebaey et al. [20] showed the benefits of using dispersed laminated composites (where the fiber angles were not limited to 0°, ±45°, an 90°) by investigating the buckling resistance and stiffness using ant colony optimisation. Similar problems were also studied by Pai et al. [21,22], using the Tabu search algorithm, and by Rama Mohan Rao et al. [23], using the scatter search algorithm. In some more recent studies, Ho-Huu et al. [24] investigated the buckling load maximization problem using the improved differential evolution and smoothed finite element method. Vosoughi et al. [25,26] showed that the fundamental frequency of a thick laminated composite plate is highly sensitive with respect to the fiber orientations. A mixed implementable evolutionary algorithm was used in order to find the fiber orientations that maximize the fundamental frequency and the buckling load. Le-Manh and Lee [27] carried out a study to maximize the post-buckling strength of a composite laminate under transverse loading for a specified amount of displacement, using a genetic algorithm with fiber orientations as the design variables. Führer [28] presented a method of progressive failure analysis for large structures, called progressive stiffness degradation analysis (PSDA). PSDA is a technique that analyses structural behaviour using closed form solutions for buckling onset and stress-based failure criteria, which significantly reduces the computational effort. In the literature [28], the PSDA method was compared to non-linear finite element analysis using Abaqus on a rectangular laminated composite plate with the stacking sequence of [45, −45, 0, 90, 0, 90, 0, −45, 45]. Analogous to the laminated plates fabricated from fiber reinforced composites, laminated glass plates also exhibit a similar structural behaviour under in-plane compression and out-of-plane bending, due to their high slenderness [29,30,31]. Buckling and delamination are common failure modes associated with both fiber reinforced composite plates and laminated glass plates. The focus of the current paper is the maximization of the buckling load for rectangular dispersed laminated composite plates using the harmony search algorithm, where the design variables are the fiber orientations and the ply thicknesses. In addition to the commonly used carbon fiber reinforced polymers (CFRP) composite material, boron/epoxy and glass/epoxy materials are also investigated for three different plate geometries. Even though not as frequently used in the structural applications as CFRP composites, plates made of the boron/epoxy composites have been shown to perform significantly better than their CFRP counterparts. Furthermore, using the harmony search algorithm, it was possible to obtain dispersed stacking sequences that exhibit higher buckling loads compared to the commonly used 0°, ±45°, and 90° stacking sequence from the authors of [28] for all of the analyzed materials and geometries.

## 2. Methods

**Figure 1:**
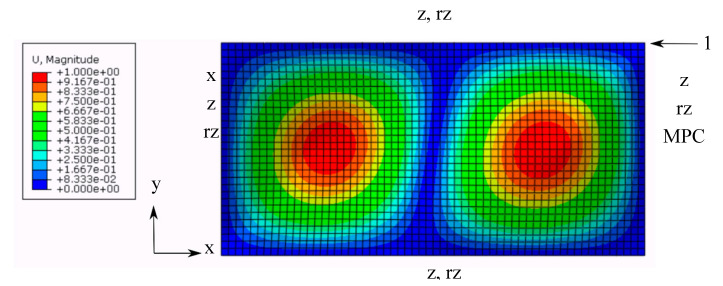
Boundary constraints for the buckling analysis.

Three different materials (carbon fiber, boron fiber, and glass fiber) were simulated. Furthermore, for each of these materials, plates with three different aspect ratios (the ratio of the longer side length to the shorter side length) were modelled and optimised with the harmony search algorithm. The fiber orientation angles of the layers and layer thicknesses of the laminated plate were chosen as the input parameters of the optimisation. For each material and each aspect ratio, the obtained maximum buckling loads were compared to the buckling load of a plate having an identical aspect ratio and a material with a stacking sequence of [45, −45, 0, 90, 0, 90, 0, −45, 45], which was adopted in the literature [28]. The buckling loads were computed using the eigenvalue buckling estimation procedure of the finite element analysis software Abaqus. In this procedure, a rectangular plate is meshed with the reduced integration shell element S4R, which is capable of modelling the bending behaviour of the composite plates. This general-purpose shell element is suitable for the eigenvalue buckling analysis, as computational performance is not a major issue [32]. The constrained degrees of freedom at each side of the plate are shown in Figure 1, where rx, ry, and rz denote the rotational degrees of freedom about the x, y, and z axes, respectively, whereas x, y, and z denote the translational degrees of freedom.

A unit concentrated force was acting on the upper right corner of the plate, as shown in Figure 1. Multi-point constraints (MPC) were applied on the right-hand side of the plate, such that all of the nodes on this side went through the same amount of displacement as the upper right corner of the plate, where the concentrated force was acting, as this force was increasing incrementally.

### The Harmony Search Optimisation Process

The application of meta-heuristic optimisation algorithms in science and engineering has significantly increased in recent years. One of the most successful and well-established techniques in this field is the harmony search technique. The harmony search algorithm has been employed for the optimum design of truss systems [33,34], steel frames [35], plate girders [36], cylindrical reinforced concrete walls and beams [37,38], plane stress systems [39], PID controlled active tuned mass damper [40], retaining walls [41], and for the stacking sequence optimisation of laminated composite plates [9]. 

The harmony search algorithm was developed by Geem et al. [42], and has been widely adopted for the optimisation of a water network design [43], a slope stability analysis [44], heat and power systems [45], job shop scheduling [46], team orienteering [47], and vehicle routing [48]. The method was initially designed with discrete valued data for musical composition, and was then further developed for application in the optimisation of continuous valued solution vectors, e.g., those encountered in the dimensioning of structural components. A parameter-setting-free version of the harmony search algorithm was developed by Geem and Sim [49]; this algorithm is more accessible and efficient, considering the difficulties associated with the proper selection of algorithm-specific parameters.

The harmony search optimisation algorithm requires a predetermined number of design variables and an objective quantity to be maximised or minimised. The design variables of a rectangular plate with a fixed aspect ratio are the thicknesses ($t_{i}$) and the ply orientation angles ($\theta_{i}$). The objective quantity to be maximised is the buckling load. The harmony search optimisation process starts with the generation of a certain number of design variable combinations, each of which is called a candidate solution vector. This initial population of candidate solutions was randomly generated within the predefined design constraints. From any given population of solutions, the solution vectors that delivered the best and worst results were identified. In the next step, based on certain rules, a new candidate solution was generated and compared to the members of the previously generated population. If the new candidate solution performed better than the worst performing solution vector in the population, the newly generated solution vector was incorporated into the population, and the previous worst-performing solution vector was removed from the population. This procedure was repeated for a predetermined number of iterations, and the convergence of the result was observed [50].

**Figure 2:**
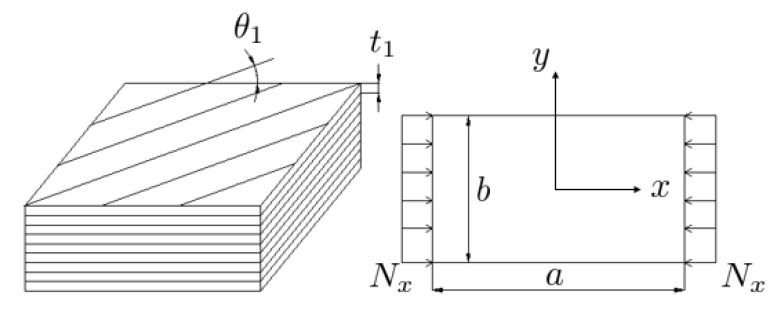
The geometry of a laminated plate with nine layers.

The buckling loads of rectangular laminated plates with nine layers [28] made of fiber reinforced composite materials were maximised in this process. Buckling under uniaxial compressive loading was considered in all of the simulations. The fiber angles and ply thicknesses of a laminated plate constituted a design vector of 18 variables. Ten of these design vectors built a population that was initialized with random values within the design constraints. The geometric configuration of a laminated plate in the optimisation process is illustrated in Figure 2. In this illustration, $\theta_{1}$ and $t_{1}$ are the fiber orientation angle and the thickness of the top layer of the nine-layered plate, respectively. Furthermore, the long and short sides of a plate are denoted with the letters a and b, respectively, in the top view of a plate on the right side of Figure 2. The unidirectional distributed load, $N_{x}$, wss applied with the help of a concentrated unit load at the lower right corner of the plate. Afterwards, all the remaining nodes on the right edge in the finite element model of the plate were constrained in such a way that they made the same amount of displacement as the lower right corner of the plate as the unit load was being increased by the load multiplier. The displacement of the boundary nodes at the left edge of the plate were constrained in the x-direction, whereas the rest of the nodes in the system were free to move in the x-direction.

In the harmony search optimisation process, the fiber angles and thicknesses of each ply could vary as the continuous variables between predefined ranges. To abide by the fabrication constraints, ply thicknesses are not allowed to be less than 0.1 mm. Furthermore, design vectors with total plate thicknesses greater than 2.25 mm were not considered as valid design options. No upper bound was defined for the thickness of an individual ply, which allowed for an optimum distribution of the ply thicknesses. The fiber angles ($\theta_{i}$ for the i-th ply in Figure 2) were allowed to vary from −90° to 90° as continuous variables. Each design vector in the population of ten different plate configurations corresponded to a buckling load level, and the next step was the computation of these buckling loads using eigenvalue buckling analysis (Abaqus). 

## 3. Results

The maximum buckling loads that we obtained from the harmony search algorithm were compared to the buckling loads of the plates with the stacking sequence given in the literature [24]. While modelling the plates with the stacking sequence of the authors of [28], the ply thicknesses were defined in such a way that all of the plies had an equal thickness and the total plate thickness was equal to the optimum plate thickness obtained from the harmony search algorithm. In the following plots of the best and worst solutions obtained from the harmony search algorithm, the aspect ratio of the plate was denoted with a/b, where a and b stand for the long and short sides of the plate, respectively. For each load case, the harmony search iterations were repeated until the best buckling load obtained from the harmony search algorithm exceeded the buckling load of the plate with the stacking sequence of the authors of [28].

### 3.1. CFRP Plates

The material properties in the finite element models of the carbon fiber plates are given in Table 1, where E1 and E2 are the elasticity moduli of a lamina in the directions parallel and perpendicular to the longitudinal axis of the fibers, respectively. G12 is the shear modulus and *ν*12 is the Poisson ratio.

**Figure 3:**
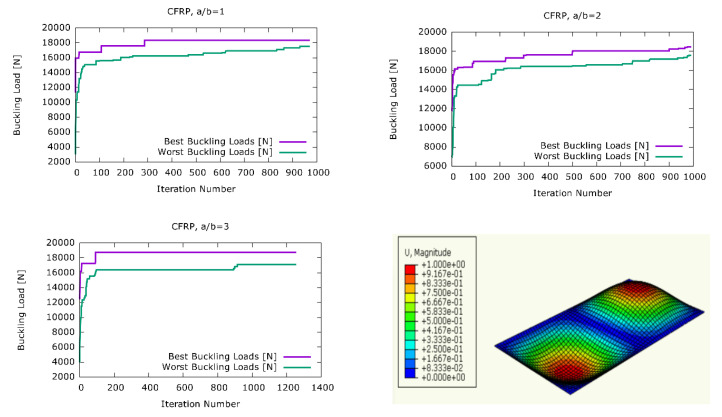
The development of the best and worst solutions in the harmony search optimisation for boron/epoxy plates.

Figure 3 shows the visualization of the harmony search optimisation stages for three different aspect ratios, in the case of the carbon fiber material together with the first buckling mode of a plate with a/b = 2, where U denotes the displacement. After each iteration, the design vectors constituting the population of stacking sequences were ranked according to their corresponding buckling loads. Afterwards, the design vectors with the highest (best) and lowest (worst) corresponding buckling loads were selected. In Figure 3, the highest and lowest buckling loads were plotted after each iteration. It can be observed that in each one of the plots in Figure 3, the value of the highest buckling load increased rapidly in the beginning, and reached its highest level after a certain number of iterations. The highest buckling load value tended to either stay at that level for the rest of the harmony search iterations, or it experienced only minor increases. Similarly, the lowest buckling load values improved rapidly in the beginning. Throughout the optimisation process, the sizes of the improvement steps for the worst buckling load tended to get smaller. However, these lowest buckling load values were expected to get closer to the best buckling loads as the number of iterations increased. Table 2 shows a summary of the results obtained from the harmony search optimisation of the CFRP plates. The total plate thicknesses of the plate configurations were 2.25 mm, 2.247 mm, and 2.248 mm for the aspect ratios of a/b = 1, a/b = 2, and a/b = 3, respectively. For the aspect ratio of a/b = 1, the best stacking sequence was [−27/43/−64/62/31/−55/57/43/48] degrees for the fiber orientation angles and [0.1/0.18/0.33/0.1/0.76/0.3/0.1/0.25/0.14] mm for the ply thicknesses. It can be observed that the mid-layers of this best stacking sequence tended to be thicker than the outer layers for the a/b = 1 aspect ratio. A similar trend can also be observed for the aspect ratio of a/b = 2, where the optimum ply thickness sequence was [0.11/0.4/0.2/0.84/0.1/0.12/0.1/0.11/0.28] mm. Here, the greatest ply thickness was observed at the fourth layer from the top as 0.84 mm. However, the remaining ply thicknesses were distributed irregularly, which implies that there was no clearly observable correlation between the thickness of a ply and its position in the stack for this load case. The irregular ply thickness distribution of [0.13/0.35/0.33/0.1/0.34/0.32/0.13/0.23/0.32] for a/b = 3 also confirms this observation. The obtained maximum buckling load of 18,337 N was 2.17% higher than the buckling load corresponding to a CFRP plate with the same aspect ratio and the stacking sequence from the literature [28]. Also, for a/b = 2 and a/b = 3, the highest buckling loads obtained from the harmony search optimisation were 1.59% and 4.5% higher, respectively, than the buckling loads of a plate with the same material properties and aspect ratio, but with the stacking sequence from the literature [28].

### 3.2. Boron/Epoxy Plates

The mechanical properties of the boron/epoxy plates are listed in Table 3. From Table 3 and Table 1, it is clear that boron/epoxy composite has superior mechanical properties compared with CFRP.

**Figure 4:**
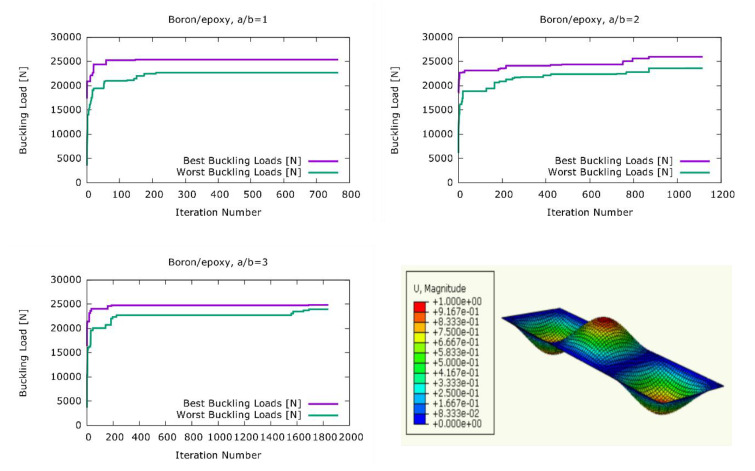
The development of the best and worst solutions in the harmony search optimisation for boron/epoxy plates.

Because of the greater elasticity moduli of boron/epoxy, plates made of this material exhibited greater buckling loads for all aspect ratios as listed in Table 4. Furthermore, similar to the load case with CFRP, the buckling loads obtained from the optimised stacking sequences were 4.55%, 5.67%, and 1.84% greater than the buckling loads obtained from plates with the stacking sequence of the literature [28] for the a/b = 1, a/b = 2, and a/b = 3 aspect ratios, respectively. As listed in Table 4, again, no clear correlation could be observed between the thickness of a ply and its position in the stacking sequence. Figure 4 shows the development of the highest and lowest buckling loads during the harmony search optimisation process. For the aspect ratios of a/b = 1 and a/b = 3, in Figure 4, the harmony search algorithm quickly reached the stacking sequence with the maximum buckling load. On the other hand, for the aspect ratio of a/b = 2, a much greater number of iterations were needed for the algorithm to settle at a maximum buckling load value.

### 3.3. Fiberglass Plates

**Figure 5:**
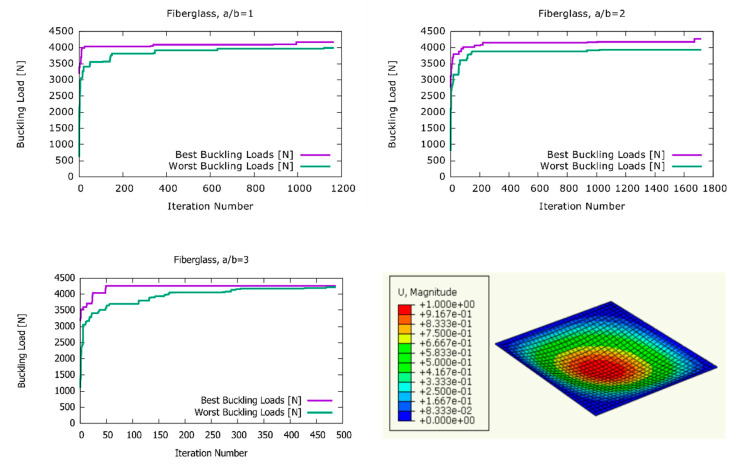
The development of the best and worst solutions in the harmony search optimisation for fiberglass plates.

As fiberglass composites have smaller elasticity moduli compared with CFRP and boron/epoxy, as shown in Table 5, the plates made of this material exhibited smaller buckling loads. The results of the harmony search optimisation for the plates made of fiberglass composite are listed in Table 6 and Figure 5. The comparison of results with the buckling loads obtained from the plates with the stacking sequence of the literature [28] showed that the buckling loads of the optimised plates were 2.43%, 4.2%, and 3.03% greater for the aspect ratios of a/b = 1, a/b = 2, and a/b = 3, respectively.

## 4. Discussion

**Figure 6:**
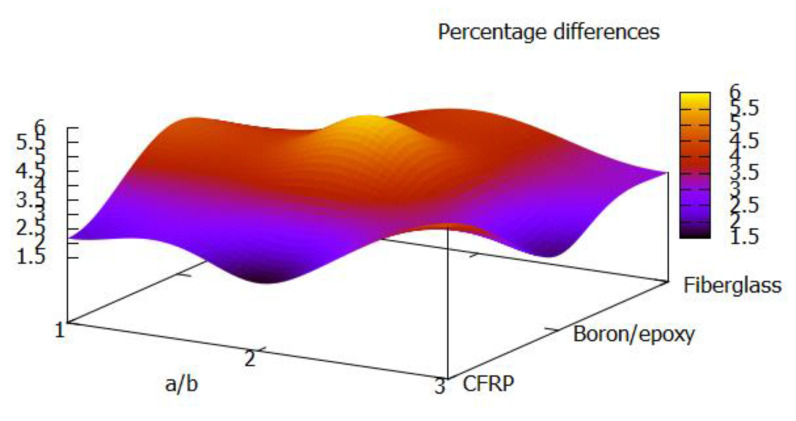
Percentage difference between the buckling loads of the optimised plates and the plates with the stacking sequence from [28].

Using the harmony search algorithm, the ply angle and thickness sequences of the laminated composite plates were optimised for three different materials and aspect ratios. The optimised plate configurations were observed to have higher buckling loads compared with a commonly used plate configuration with a [45°, −45°, 0°, 90°, 0°, 90°, 0°, −45°, 45°] ply angle sequence. Figure 6 shows that the optimised plate configurations performed up to 5.67% better than the plate configuration used in the literature [28]. This highest performance improvement was achieved with a boron/epoxy plate with an aspect ratio of 2. The corresponding ply angle sequence was [49°/64°/−32°/−52°/21°/−82°/−3°/49°/−47°], which shows that the structural performance could be enhanced through the introduction of irregular ply angles into the stacking sequence.

**Figure 7:**
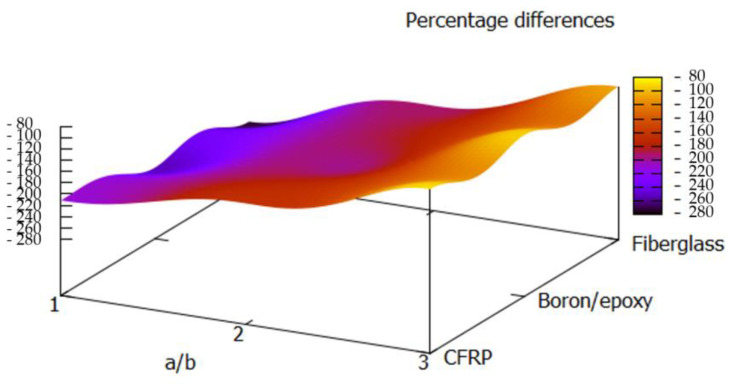
Percentage difference between the buckling loads of the optimised plates and the plates with the stacking sequence from [52].

To demonstrate this improvement in structural performance on a different plate configuration and to compare the performances of the optimised configurations with more than one reference, another stacking sequence proposed by Muc [52] with the ply sequence of [0°, ±15°, ±30°, ±45°, ±60°, ±75°, 90°] and equal ply thicknesses was analysed. Figure 7 shows the percentage differences between the optimised configurations and the plate configuration from the literature [52]. It can be observed that the harmony search optimisation technique delivered buckling loads of on average 176% and up to 254% greater buckling loads compared with the lay-up proposed in the literature [52]. This observation indicates once again that choosing the right stacking sequence can have a profound impact on the structural performance.

Further research in this field could be carried out with hybrid plate configurations using different types of composite reinforcement in the core layers and the outer layers. It is known that materials with lower strength and stiffness properties can be used in the core layers of hybrid composites without reducing the overall structural performance [53,54]. Therefore, incorporating the stiffness and strength properties of the layers as additional design variables of optimisation can lead to better design with a lower cost. In addition to fiber reinforced composites, laminated glass panels constitute another type of structural member, which is prone to buckling because of its high slenderness [29,30,31]. Therefore, future research in this field could include the optimisation of laminated glass panels with respect to various material and geometrical properties using the harmony search algorithm.

## 5. Conclusions

Composite materials are increasingly applied in structural members because of their better strength and stiffness properties and lower weight compared with traditional structural materials. Particularly carbon fiber reinforced polymers (CFRP) have found widespread application in structural systems, while other composite materials such as boron/epoxy and glass/epoxy have received less attention from the researchers. In this study, we used a well-established metaheuristic optimisation algorithm called the harmony search algorithm to obtain the stacking sequence for a dispersed laminated composite plate that delivers the maximum buckling load under certain thickness constraints. For three different plate aspect ratios, the stacking sequences obtained from the harmony search optimisation delivered buckling loads greater than what the stacking sequence from the literature [28] delivers. This result indicates that it is possible to obtain a better performance from laminated composites using dispersed configurations, albeit the stacking sequences with fiber angles fixed at 0°, ±45°, and 90° are commonly applied in the industry.

The comparison of the maximum buckling loads of CFRP, boron/epoxy, and glass/epoxy composite plates after the optimisation of the stacking sequences showed that boron/epoxy plates exhibit the best performances because of the superior mechanical properties of the boron/epoxy composites. In practical applications, most of the time the ply thicknesses are kept constant among all layers, as to the best of the authors’ knowledge, there is no well-established ply thickness distribution pattern that performs better than the constant ply thickness distribution. As a result of this condition, the availability of meta-heuristic optimisation algorithms, such as harmony search, is a great advantage in order to discover better ply thickness distributions on a case by case basis. This study also showed that it is possible to obtain ply angle sequences that perform better than the 0°, ±45°, and 90° sequence commonly applied in the industry. Therefore, it is crucial for design engineers to have access to optimisation algorithms like harmony search, as these algorithms can deliver the best performing stacking sequence specific to any given material and geometry configuration. 

## Figures and Tables

**Table 1:** Material properties of carbon fiber reinforced polymers (CFRP) plates.

Material Property	Carbon Fiber (CFRP) [28]
E1 [N/mm^2^]	157,000
E2 [N/mm^2^]	8500
G12 [N/mm^2^]	4200
ν12	0.35

**Table 2:** The outcome of the harmony search optimisation for the CFRP plates.

Material Property	a/b = 1	a/b = 2	a/b = 3
Total plate thickness (mm)	2.25	2.247	2.248
Max buck. load HS (N)	18,337	18,446	18,722
Max buck. load Führer [28] (N)	17,947	18,157	17,916
Best stacking sequence (degrees)	[−27/43/−64/62/31/−55/57/43/48]	[56/−48/58/60/31/66/40/51/−50]	[46/−40/55/29/80/3/73/46/−43]
Best ply thicknesses (mm)	[0.1/0.18/0.33/0.1/0.76/0.3/0.1/0.25/0.14]	[0.11/0.4/0.2/0.84/0.1/0.12/0.1/0.11/0.28]	[0.13/0.35/0.33/0.1/0.34/0.32/0.13/0.23/0.32]

**Table 3:** Material properties of boron/epoxy plates.

Material Property	Carbon Fiber (CFRP) [28]
E1 (N/mm^2^)	207,540
E2 (N/mm^2^)	19,790
G12 (N/mm^2^)	5520
ν12	0.225

**Table 4:** The outcome of the harmony search optimisation for the boron/epoxy plates.

Material Property	a/b = 1	a/b = 2	a/b = 3
Total plate thickness (mm)	2.239	2.249	2.242
Max buck. load HS (N)	25,395	26,000	24,854
Max buck. load Führer [28] (N)	24,289	24,606	24,406
Best stacking sequence (degrees)	[−47/51/−29/−38/61/55/86/42/−38]	[49/64/−32/−52/21/−82/−3/49/−47]	[64/39/3/49/−43/−47/−50/44/−43]
Best ply thicknesses (mm)	[0.12/0.43/0.16/0.46/0.21/0.1/0.14/0.37/0.26]	[0.22/0.1/0.1/0.28/0.11/0.12/0.32/0.66/0.34]	[0.1/0.1/0.11/0.1/0.22/0.1/1.03/0.29/0.2]

**Table 5:** Material properties of fiberglass plates.

Material Property	Fiberglass [51]
E1 (N/mm^2^)	33,000
E2 (N/mm^2^)	3100
G12 (N/mm^2^)	3000
ν12	0.26

**Table 6:** The outcome of the harmony search optimisation for the fiberglass plates.

Title 1	a/b = 1	a/b = 2	a/b = 3
Total plate thickness (mm)	2.244	2.246	2.248
Max buck. load HS (N)	4168	4270	4254
Max buck. load Führer [28] (N)	4069	4098	4129
Best stacking sequence (degrees)	[−53/34/49/20/61/−76/−29/38/−45]	[41/50/−50/−16/−83/40/62/−51/−34]	[49/−47/68/45/−66/60/52/−36/−42]
Best ply thicknesses (mm)	[0.32/0.1/0.22/0.1/0.11/0.78/0.1/0.42/0.1]	[0.1/0.17/0.43/0.1/0.45/0.63/0.1/0.16/0.1]	[0.2/0.26/0.38/0.26/0.1/0.3/0.42/0.1/0.22]
